# Mr. Edwards's Case of Inflammation of the Intestines

**Published:** 1810-01

**Authors:** G. F. Edwards

**Affiliations:** Member of the Royal College of Surgeons, &c. London. Bath


### *To the Editors of the Medical and Physical Journal.*

GENTLEMEN,

LATE in the evening of the 31st of October, I was requested to visit Mr. A. of this city, who was seized with a violent pain in the region of his liver: the above was the message of the servant. This gentleman had been some time in a warm climate, and had suffered much from a liver complaint; and, since his return, very frequently endured severe cramp-like pains from that cause, which very naturally led him to believe, that the present pain was of the same nature with the past, and caused by the same means. In order to ease this uneasiness, he took liberally of brandy, as he always found it give him instantaneous relief: but, on this occasion, he was disappointed. As the pain increased, it alarmed his wife; and, in consequence, I was applied to. When I first saw him, he complained of a violent acute pain on the right side of his bowels, which increased and diminished by fits; but had left that side, he said, since he drank the brandy, and gone to the left; and, at times, the pain appeared to occupy the whole abdomen. I could gain no satisfactory information when his bowels were evacuated; but I knew they were generally constipated, which naturally arose from the imperfect action of the liver, and the bile getting access to the stomach, instead of finding its way into the intestines by its own common duct. His pulse was small, hard, and frequent, 120 in a minute. Considering that there was obstruction — though no particular evidence being present — calomel and cathartic extract were given, with the view of acting smartly on the bowels; and a considerable quantity of blood taken from the arm—warm fomentations to the whole abdomen. Notwithstanding this treatment, the pain increased with considerable siderable tension, especially on that part corresponding with the sigmoid flexure of the colon. A cathartic injection was thrown per anum; and a cathartic mixture, composed of infus. sennæ ℥vß; tinct. sennæ ℥ß; magnes. sulphas ℥j. Two spoonfuls every hour were ordered, till evacuations should be produced; but these remedies only served to aggravate the disease. His strength began to fail, and profuse perspiration poured from every pore. In this state I felt satisfied he could not long remain alive, therefore further advice was called in; and Dr. Murray, an able physician of this city, did me the honour to say, that I had pursued every step that could be taken, which must be continued, till the desired end of removing the constipation was effected. All the means of resolution were persisted in with unabated zeal, but all to no purpose; as the disease baffled every endeavour which judgment or prudence could suggest; and we were witnesses to the defeat of all our projects, by the untimely fate of our patient, who was cut off in less than twenty-four hours from the first attack.

*Appearances on Dissection.*

After making an incision through the skin and cellular membrane, and clearing away the abdominal muscles, the first morbid appearance presenting itself was slight peritoneal inflammation, particularly on the left side, the seat of the pain. The peritoneum being divided, all present were struck with the brilliant appearance (if I may be allowed the expression) the whole surface of the intestines presented, of a fine crimson colour; but the transverse arch of the colon, the sigmoid flexure, and that portion of the ileum terminating in the caput coli, bore strong marks of violent inflammation; as they were most particularly red, and some of the vessels distended, and very large. A considerable quantity of coagulable lymph was thrown out upon these parts so highly inflamed, with here and there a few spots of extravasated blood; which was chiefly confined to about three inches of the sigmoid flexure of the colon. The inferior portion of the duodenum contained a considerable quantity of indurated matter; the rectum, also, was full of hard fæces; but no other part of the intestines. The cavity of the abdomen contained about three pints of a yellow limpid fluid; the liver bore evident marks of disease, being very dark, of a deep chocolate colour, firm in texture, and much enlarged.

The remaining abdominal viscera were all natural; the stomach empty, and externally exhibited no morbid appearance. The vesica fellis was distended with gall; and the urinary bladder, by some accident, was ruptured: consequently, had not an opportunity of examining it. As this gentleman had no thoracic complaint, we did not conceive it necessary to open the chest.

### Remarks.

The objects most worthy notice were: 1. The sudden attack of pain, and the transition of that pain from the right to the left side. 2. The rapid progress and extent of inflammation; and, 3. The nature of the obstruction.

When I reflect that my patient considered himself as well as usual when at supper with his family, and in a moment to be seized with such sudden violent pain, and that pain to shift to the opposite side, and produce such active inflammation as to destroy life, at first sight appears strange; but when I reflect also, that this gentleman laboured under liver complaint, and suffered frequent attacks of violent pain, which he never failed to relieve with spirits, and that his bowels were always in a confined state, wonder ceases. I conceive the first pain that he felt was in the pylorus, probably caused by the increased pressure made by a distended stomach, upon the already distended and obstructed duodenum. This pain was relieved by the spirits which he took liberally; and caused the morbid action to be determined at a greater distance from the obstruction.

2. The rapid progress made by the inflammation, and its extensive influence, may, in a measure, be satisfactorily accounted for. I conceive the habit was saturated with inflammatory materials, and only wanted a principal agent, such as partial obstruction, &c. to commence the fatal work; in which I am more confirmed, by the appearance of the liver carrying its own evidence, of having been acted upon by the stimulus of ardent spirits.

3. The nature of the obstruction certainly was no more than a secondary cause of difficulty; this is sufficiently clear, I think, from the stomach not sympathising with the general inflammatory action, as it kept every thing upon it which was offered. I am of opinion, that the reason why none of the very active means employed, did not pass the alimentary canal, was, that the violence of the inflammatory action prevented the natural peristaltic motion motion of the intestines. If we do not admit this, and if the obstruction in the duodenum was the cause of all the difficulty, would not my patient have felt constant pain in that part; and would not the stomach have sympathised with, and rejected every remedy offered to remove that obstruction: but we find the reverse of the above to have taken place. As the pain being relieved, which was very evidently about the situation of the obstruction, and that pain attacking the sigmoid flexure of the colon, a considerable distance from the duodenum, which explains why the stomach remained so quiet, even until death; which leaves no doubt on my mind, as to the nature of the disease and cause of dissolution, viz. violent and extensive inflammation, excited by the constant use of ardent spirits. I am, &c.

G. F. EDWARDS,
Member of the Royal College of Surgeons, &c. London.

*Bath, Dec.* 1809.

